# Source and succession of microbial communities and tetramethylpyrazine during the brewing process of compound-flavor Baijiu

**DOI:** 10.3389/fmicb.2024.1450997

**Published:** 2024-08-06

**Authors:** Wei Cheng, Wei Lan, Xuefeng Chen, Xijia Xue, Huipeng Liang, Huawei Zeng, Ruilong Li, Tianquan Pan, Na Li, Hongwen Yang

**Affiliations:** ^1^School of Biology and Food Engineering, Fuyang Normal University, Fuyang, Anhui, China; ^2^Technology Center of Enterprise, Anhui Jinzhongzi Distillery Co., Ltd., Fuyang, Anhui, China; ^3^School of Food Science and Engineering, Shaanxi University of Science and Technology, Xi'An, Shaanxi, China; ^4^Technology Research Institute, China Resources Snow Breweries Co., Ltd., Beijing, China; ^5^School of Life Sciences, Huaibei Normal University, Huaibei, Anhui, China

**Keywords:** compound-flavor Baijiu (CFB), fermented grains (FG), microbial community, pit mud (PM), volatile compounds

## Abstract

Pyrazines are important flavor components and healthy active components in Baijiu, which including tetramethylpyrazine (TTMP). During the brewing process, the traceability of microbial communities and the content distribution characteristics of TTMP are important for improving the quality and style characteristics of compound-flavored Baijiu (CFB). However, the traceability analysis of microorganisms in fermented grains (FG)—used in the production of CFB—lacks quantitative and systematic evaluation. In this study, the microbial communities and TTMP content of Jiuqu (JQ), Liangpei (LP), FG, and pit mud (CP) used in CFB production were characterized; further, coordinate and discriminant analyses were employed to determine differences in microbial communities. Additionally, traceability and correlation analyses were performed to reveal the origin of microbial communities in FG. The source, content, and distribution characteristics of TTMP based on the brewing process have also been discussed. The results showed that most of the bacterial and fungal communities at different levels of FG came from other sources, and the microorganisms of *Cladosporium, Acetobacter, Aspergillus, Methanosarcina*, and *Bacillus* were considered have a osculating correlations with TTMP content of FG. Taken together, this study provides insights into the origin of microbial communities in FG and the distribution characteristics of TTMP based on the CFB brewing process. The current findings are conducive for optimizing the fermentation process and improving the quality and style characteristics of CFB.

## 1 Introduction

As one of the six major distilled liquors worldwide, Baijiu is preferred by the Chinese people for its unique flavor and cultural significance (Tu et al., 2022). Baijiu is mainly produced via traditional solid-state fermentation, which includes Jiuqu (JQ; starter for fermentation) production, grain steaming, mixed fermentation, distillation, blending, maturation, and other processes (Ye et al., 2021). Baijiu fermentation is an intricate bilateral fermentation process involving multiple strains of microorganisms, including bacteria, yeasts, and molds, with subtle interactions between microbial communities, physicochemical indices, and environmental factors creating a unique brewing microecology in which microorganisms play a key role (Jin et al., 2017; Chai et al., 2021; Wang et al., 2021, 2023; Huang et al., 2022). The microbial communities of Baijiu during brewing are affected by various factors, such as the raw materials used, JQ, the fermentation cellar, and the brewing environment, among which JQ is the main source of microbial communities during fermentation (Wang et al., 2017; Zou et al., 2018; Tan et al., 2019). Source tracking analysis of microbial communities during the brewing process of strong-flavor Baijiu indicates that approximately 10–20% of the bacterial communities and 60–80% of the fungal communities originate from JQ, with the microbial community succession rate imparting flavor differences (Wang et al., 2017; Guan et al., 2020). Furthermore, approximately 80% of the carbohydrate hydrolases during Baijiu fermentation are from JQ, mainly derived from *Aspergillus, Rhizomucor*, and *Rhizopus* (Wang et al., 2020). But for the brewing of CFB, the main source of microbial communities in fermented grains (FG) from different process stages are still unknown, which need to be studied to adjust the brewing process. Similar to source-tracking analysis, fast expectation-maximization microbial source tracking (FEAST) can simultaneously estimate the contribution of thousands of potential source environments (Shenhav et al., 2019).

Flavor components are essential factors that determine the quality and style of Baijiu, although they comprise ~2% of trace components (Hong J. et al., 2021; Wang et al., 2022). Owing to differences between regions, brewing technology, and raw materials, a variety of Baijiu styles have emerged, exhibiting different flavor characteristics (Jin et al., 2017; Hong J. et al., 2021; Wang et al., 2022). The microbiota and metabolites are important factors in the production of Baijiu. Microbial flavor metabolism in solid-state fermentation is a complex mixture of microorganisms, trace compounds, and enzymes, which is influenced by raw materials, pit mud, and environmental factors (natural microflora, air, and water) (Jin et al., 2017).

Based on flavor characteristics, Baijiu can be classified into 12 major flavor types, including jiang flavor, strong flavor, light flavor, rice flavor, and other flavor types derived from these (Tu et al., 2022; Wang et al., 2022). Compound-flavor Baijiu (CFB) is derived from two or more of the above-listed four main flavors and exhibits complex aroma profiles due to unique brewing technologies, including high-temperature Daqu (HDQ), medium-temperature Daqu (MDQ), and Fuqu (FQ), which are applied during the brewing process (Cheng et al., 2022, 2023a; Dan et al., 2024). Tetramethylpyrazine (TTMP), a special flavor substance with pharmacological effects, imparts a nutty and baked aroma to Baijiu, along with health benefits, thus impacting its flavor and quality (Shi et al., 2022). TTMP can be produced via the Maillard reaction and microbial synthesis during Jiuqu-making, stacking fermentation, and solid-state fermentation of Baijiu (Starowicz and Zieliński, 2019; Shi et al., 2022). Similar to microbial traceability analysis, tracing TTMP content during the brewing process is important for the quality improvement of CFB. Summary, the sources of fermentation microbiota and the content changes of TTMP during CFB fermentation are not well understood. Especially, the association between functional microorganisms and TTMP content are need to research further.

In this study, the microbial communities and TTMP content of HDQ, MDQ, FQ, Liangpei (LP), different depths of fermented grains (FG), and pit-mud (CP), which are involved in CFB production, were detected. Further, coordinate and discriminant analyses were employed to characterize the differences in microbial communities among the groups. Traceability and correlation analyses were also used to determine the origin of microbial communities in FG. Additionally, the source, content, and distribution characteristics of TTMP were assessed, which are conducive to optimizing the fermentation process and improving the quality and style characteristics of CFB.

## 2 Materials and methods

### 2.1 Sample collection

All samples were collected from the brewing workshop of Anhui Jinzhongzi Distillery Co., Ltd. (Hebin Street No.230, Fuyang, Anhui 236023, P. R. China). A five-point sampling method was employed to select JQ samples from the workshop, including HDQ, MDQ, and FQ, which were then ground into powder. LP samples were collected from the surface and inner parts of the stacking-fermentation grains using a five-point sampling method after 48 h of stacking-fermentation and were then mixed in equal quantities.

FG samples were collected from the upper, middle, and lower levels of the fermentation cellar using a five-point sampling method after 65 days of fermentation and were termed CA (from the upper levels of FG), CM (from the middle levels of FG), and CB (from the lower levels of FG), respectively. Five diagonal samples were collected from the bottom of the fermentation cellar using a five-point sampling method and mixed as parallel CP samples (Cheng et al., 2024).

All samples were packed in sterile self-sealing bags, and each sample was divided into two parts. One was stored at −80°C for total DNA extraction, the other was stored at 4°C for the detection of TTMP. All experiments were performed in triplicate.

### 2.2 High-throughput sequencing

Pretreatment and DNA extraction were performed as described by Xu et al. (2023). For bacteria, the V4 region of the 16S rRNA gene was amplified using the universal primer sets 515F^c^ (5′-GTGYCAGCMGCCGCGGTAA-3′) and 806R^c^ (5′-ACGTCATCCCCACCTTCC-3′). For fungi, the internal transcribed spacer (ITS). I region was amplified with primers ITS5F (5′- GGAAGTAAAAGTCGTAACAAGG−3′) and ITS2R (5′-GCTGCGTTCTTCATCGATGC-3′). The polymerase chain reaction products were assessed using a Quanti Fluor-ST Blue Fluoroscopy Quantitative System (Promega, Madison, WI, USA). Library construction and genome sequencing were performed by Shanghai Personal Biotechnology Co., Ltd. (Shanghai, China) using a MiSeq Benchtop Sequencer (2 × 300 bp; Illumina MiSeqPE300, San Diego, CA, USA).

Raw sequences were processed using QIIME2 (2019.4) software (Illumina), and those with ≥97% similarity were classified into the same operational taxonomic unit (OTU). Representative bacterial OTU sequences were annotated using the Silva-138-116 S rRNA database, and representative fungal OTU sequences were compared with those from the Unite-9 fungal ITS database (Hong J. et al., 2021; Cheng et al., 2023b; Xu et al., 2023).

### 2.3 Bioinformatic processing pipeline

The α-diversity of microbial communities was evaluated based on the observed species and the Shannon–Wiener and Simpson indices. Bray–Curtis distance was employed to determine the differences in β-diversity among groups. Principal coordinate analysis and orthogonal partial least squares discriminant analysis (OPLS-DA) were performed on the distance matrices to visualize the relationships among groups using the vegan package version 2.5.3 (R Core Team, Vienna, Austria) and DiscriMiner package (version 0.1-29) in R software 4.0.5 (Mathsoft, Inc. USA). In addition, variable importance in projection (VIP) of the OPLS-DA model and linear discriminant analysis effect size (LEfSe) were used to differentiate biomarkers among different groups (Hong J. et al., 2021; Hong L. et al., 2021; Xu et al., 2023; Cheng et al., 2024).

### 2.4 Determination of TTMP content via headspace solid-phase microextraction coupled with gas chromatography-mass spectrometry (HS-SPME-GC-MS)

TTMP content was determined using a previously described method (Kwon et al., 2013; Hu et al., 2021; Al-Dalali et al., 2022), with slight modifications. Briefly, JQ (HDQ, MDQ, and FQ) was crushed to 70-mesh size, and LP, CA, CM, CB, and CP were then homogenized for testing. Three grams of crushed or homogenized samples were placed in a 50-mL centrifuge tube, and 30 mL ethanol solution (20%) was added. After ultrasonic extraction for 60 min and centrifugation at 7,000 rpm at 4°C for 6 min, 5 mL of supernatant was absorbed into a 20-mL headspace bottle. Thereafter, 1.6 g NaCl was added, and the bottle cap was tightened for testing.

Conditions for the automatic sampler included 50/30 DVB/CAR/PDMS (2 cm) extraction head, equilibrium at 50°C for 10 min, an extraction time of 40 min, and a stirring rate of the magnetic stirrer at 400 rpm.

For GC, a DB-5MS chromatographic column (30 m × 0.25 mm × 0.25 μm; Agilent Technologies, USA) and the following gradient conditions were used: initial temperature of 50°C for 1 min, increased to 100°C at 8°C/min for 5 min, and then increased to 235°C at 15°C/min for 4 min. Helium (purity >99.999%) with a flow rate of 2 μL/min was used as the carrier gas. Shunt sampling was performed at a shunt ratio of 5:1.

For MS, the electron bombardment energy was 70 eV, the ion source temperature was 230°C, the four-stage rod temperature was 150°C, and transmission line temperature was 245°C. Selective ion monitoring was applied with monitoring ions (m/z) 136, 54, 42, quantitative ions 136, and a solvent delay time of 5 min.

The standard curve and content calculations for the TTMP test were performed as follows:

(1) Preparation of reserve liquid (1.00 mg/mL): TTMP standard (0.05 g) was accurately weighed (accurate to 0.0001 g), dissolved with anhydrous ethanol, maintained at a fixed volume of 50 mL, and stored in a refrigerator at a low temperature of 0–4°C.
(2) Preparation of the intermediate solution (10.0 μg/mL): The TTMP reserve solution (1.00 mL) was accurately absorbed (1.00 mg/mL), the volume of anhydrous ethanol was fixed to 100 mL, and sealed and stored in a refrigerator at a low temperature of 0–4°C.
(3) Preparation of standard curve working solution: The intermediate solution (10, 20, 50, 100, 200, and 500 μL) was accurately absorbed in a 10-mL volumetric bottle with 20% anhydrous ethanol solution to scale. Thereafter, standard curve working solutions of 1.0, 2.0, 5.0, 10, 20, and 50 ng/mL were prepared.
(4) Next, 5 mL of standard curve working solutions with concentrations of 10, 20, 50, 100, 200, and 500 μL were accurately removed, 1.6 g of NaCl and a magnetic rotor were added, the cap was pressed and mixed well, and HS-SPME-GC-MS was then performed. A standard curve was drawn with the standard concentration as the horizontal coordinate and the peak area of TTMP in the standard curve working solution as the vertical coordinate.
(5) The moisture content of the samples was determined based on the Chinese standard methods (GB 5009.3-2016) (National Standard for Food Safety, 2016).
(6) Calculation of TTMP content: the sample solution was measured using a standard curve working solution, and the TTMP content in the sample was calculated. The formula is as follows:

$$\begin{array}{l} X=\;\frac{\text{c}\times \text{V}\times 1000}{\text{m}\left(1-X_{1}\right)\times 1000} \end{array}$$

Where X is the content of TTMP in the absolute dry sample (μg/kg), c is the solution mass concentration calculated according to the peak area of the sample (ng/mL), V is the constant volume of the sample (mL), m is the sample quantity (g), X1 is the moisture content in the sample (%), and 1,000 indicates the conversion factor.

### 2.5 FEAST traceability analysis of microorganisms in FG

The mean values of the relative abundance of each sample were calculated, and the sources of microbial communities in the FG (CA, CM, and CB) were predicted using the R Studio program in the R language. FEAST traceability analysis (https://github.com/cozygene/FEAST) was performed using R language Studio (Shenhav et al., 2019). The samples of HDQ, MDQ, FQ, LP, and CP were set as the ‘source end', and the samples of FG (CA, CM, and CB) were set as the ‘receiving end' for traceability analysis.

### 2.6 Traceability of TTMP

The TTMP content of the samples was determined, and the generation and migration patterns of TTMP during the CFB brewing process were assessed to provide a theoretical basis for guiding the regulation of TTMP content in CFB production, elucidating the brewing mechanism, and improving the quality of CFB.

### 2.7 Statistical analysis

All measurements were performed in triplicate and are presented as mean ± standard deviation. Duncan's test was employed to evaluate the statistical significance of differences via one-way ANOVA using SPSS v 21.0 software for Windows (IBM). Statistical analyses and graphic visualization were performed using GraphPad Prism 9 software (GraphPad Software Inc., San Diego, CA, USA), Microsoft Excel (Microsoft, Redmond, Washington, USA), and Adobe Illustrator CC 2018 (Adobe Systems Inc., SAN Jose, California, USA).

## 3 Results and discussion

### 3.1 Composition and variation of the microbial communities in different samples based on the CFB brewing process

#### 3.1.1 Venn analysis of sequencing data, α-diversity, and OTU distribution

After quality control, 116,901–147,991 bacterial 16S rRNA gene sequences and 133,241–146,926 fungal ITS gene sequences were obtained, with average lengths of 211–222 and 228–237 bp, respectively. The average number of high-quality sequences was 11,372 for bacteria and 117,805 for fungi, and the sparse curves tended to flatten (Supplementary Figure S1), indicating sufficient sequencing depth for subsequent analyses (Li et al., 2021).

**Figure 1:**
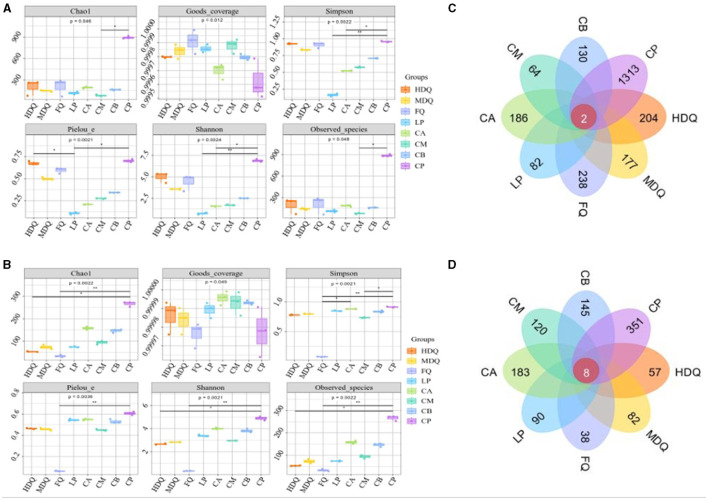
Analysis of microbial α-diversity and Venn diagram of microbe core OTU distribution of different samples. **(A)** α-diversity of bacteria; **(B)** α-diversity of fungi; **(C)** Venn diagram of bacteria; **(D)** Venn diagram of fungi.

For both bacteria and fungi, the coverage values were >0.9995 (Figures 1A, B), demonstrating the reliability of high-throughput amplicon sequences for evaluating microbial community information in all samples (Zuo et al., 2020; Li et al., 2021). Notably, for the LP group, the Shannon and Simpson indices of sequenced bacterial DNA were higher than those of the other sample groups (Figure 1A), indicating higher uniformity and diversity of bacterial species in the LP group. For the FQ group, the Shannon and Simpson indices of sequenced fungal DNA were lower than those of other sample groups (Figure 1B), indicating lower uniformity and diversity of fungal species in the FQ group.

As shown in Figure 1C, two bacterial OTUs were identified in the different sample groups, accounting for 0.08% of the total OTUs in the sample group. Among them, the number of unique OTUs in the CP group was the highest (1,313), accounting for 54.85% of the total OTUs, indicating the highest bacterial diversity in the CP group than that in the other sample groups, which was consistent with the Shannon and Simpson indices. In addition, the number of bacterial OTUs in the CM group was 64, which was significantly lower than that in other sample groups, accounting for 2.67% of the total OTUs, indicating lower bacterial diversity in the CM group, which deviated from Shannon and Simpson indices (Figure 1A), necessitating further analyses.

As shown in Figure 1D, eight fungal OTUs were identified in the different sample groups, accounting for 0.63% of the total OTUs. The maximum number of unique OTUs in the CP group was 351, accounting for 27.66% of the total OTUs, indicating the highest fungal diversity in the CP group than that in the other sample groups, which was consistent with the Shannon and Simpson indices. In addition, the number of bacterial OTUs in the FQ group was 38, which was significantly lower than that in the other sample groups, accounting for 2.99% of the total OTUs, indicating lower fungal diversity in the FQ group than that in the other sample groups, which was consistent with the Shannon and Simpson indices (Figure 1B).

#### 3.1.2 Analysis of microbial community structure at the phylum level

**Figure 2:**
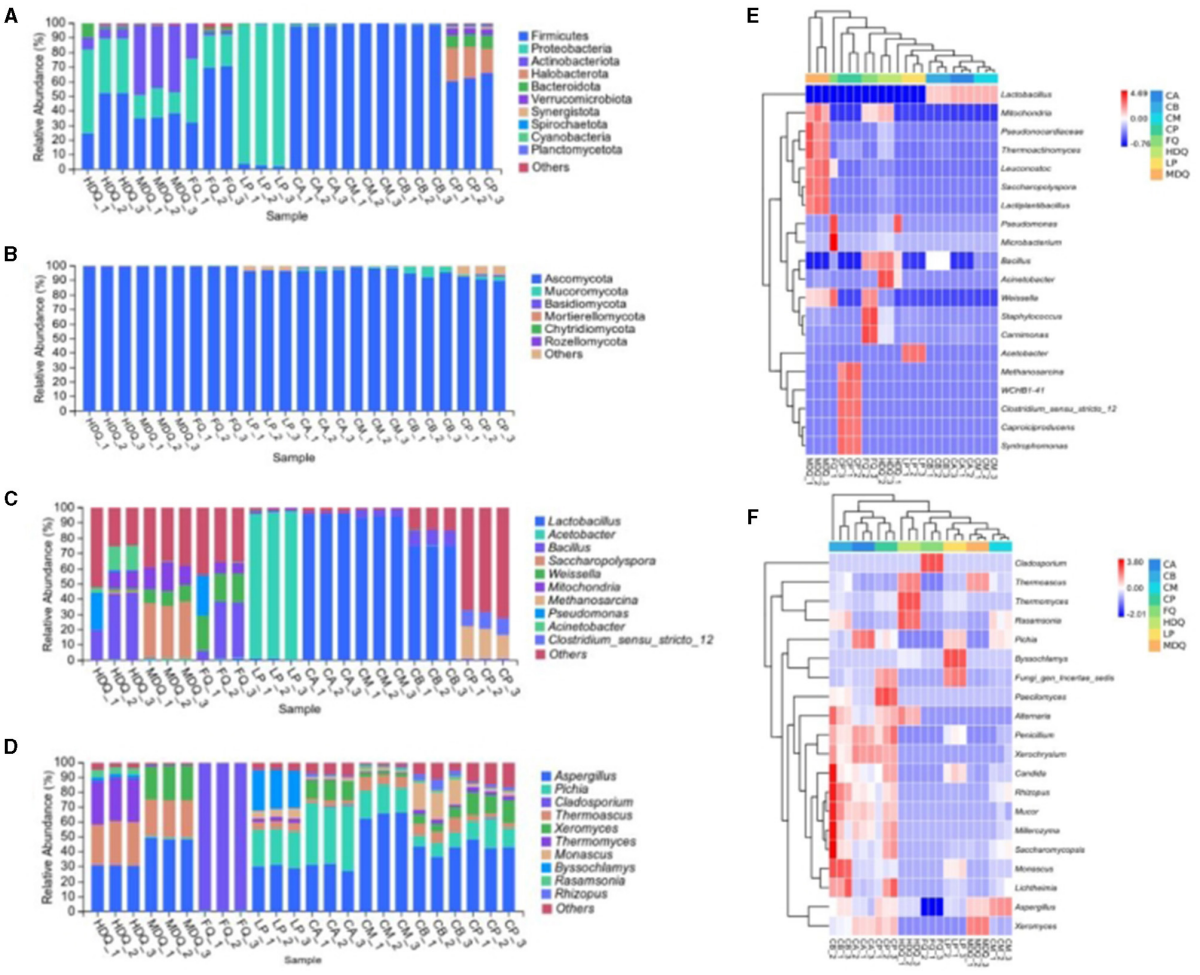
Microbial community structure at the phylum and genus level between different samples during the brewing process. **(A)** bacterial phyla; **(B)** fungal phyla; **(C)** bacterial genera; **(D)** fungal genera; **(E)** heatmap of bacteria genus; **(F)** heatmap of fungi genus.

Representative sequences of various OTUs were compared with the Unite database to obtain species classification information for each OTU at different taxonomic levels (Hong J. et al., 2021). A total of 23 bacterial phyla were detected in the samples (Figure 2A). For FQ, the relative abundances of Firmicutes, Proteobacteria, and Actinobacteria exceeded 20%. For FG (CA, CM, and CB), the relative abundance of Firmicutes was up to 99.85%. For LP, the relative abundance of Proteobacteria was the highest at 97.61%.

Notably, the relative abundances of Firmicutes, Halobacterota, Bacteroidetes, and Verrucomicrobiota in CP were >4%, which were the dominant bacterial phyla in CP but different from those in CB. During Baijiu fermentation, the bottoms of FG and CP are in contact and influence each other, with the variety and abundance of microorganisms affecting flavor (Liu and Sun, 2018; Qian et al., 2021). In our analysis, the CB bacterial community had no significant effect on the bacterial community composition of CP at the phylum level.

As shown in Figure 2B, seven fungal phyla were detected in each sample. For HDQ, MDQ, and FQ samples, Ascomycota was the dominant fungi, with a relative abundance of >90.00%. Ascomycota was also the dominant phylum for different levels of FG (CA, CM, and CB), with a relative abundance of 80.00%. For CP, Ascomycota exhibited a relative abundance of ≤ 70.00%, and Mucoromycota accounted for >7.0%. In addition, the relative abundances of unidentified bacterial and fungal phyla were 0.47% and 0.57%, respectively, which required further analysis.

#### 3.1.3 Analysis of microbial community structure at the genus level

Based on the brewing process, samples were classified into six main bacterial genera (Figure 2C): *Lactobacillus, Acetobacter, Bacillus, Saccharopolyspora, Weissella*, and *Mitochondria*, whose relative abundances were all >1%. The dominant bacterial genera in different sample groups showed significant differences. For FQ, *Bacillus* and *Weissella* were the dominant genera, with relative abundances of >18%. The relative abundance of *Lactobacillus* at different levels of FG (CA, CM, and CB) was as high as 70%, representing the dominant bacterial genus in FG.

For LP, the relative abundance of *Acetobacter* was 94.86%, representing the absolutely dominant bacterial genus, whereas *Bacillus* and *Lactobacillus* in LP had relative abundances of approximately 1.0% each, representing the dominant bacterial genera. During fermentation, *Acetobacter, Bacillus*, and *Lactobacillus* deliver enzymes and aroma, thereby providing important flavor substances and their precursors in Baijiu (Tu et al., 2022; Cheng et al., 2023a,c). Notably, the relative abundance of *Acetobacter* on the pile surface in traditional stacking fermentation was significantly higher than that in the interior, which may be related to the lower temperature of the pile surface (Cheng et al., 2023d).

Notably, the relative abundance of *Lactobacillus* in CB was ~72%, significantly lower than that in CA and CM (both approximately 94%). Meanwhile, the relative abundance of *Lactobacillus* in the CP was approximately 0.53%, that of *Methanosarcina1* in CP was approximately 15%, and that of *Clostridium_sensu_stricto_12* in CP was ~10%. In conclusion, CP had a certain effect on the bacterial composition of CB at the genus level, with noticeable differences between CP and CB.

The relative abundances of the *Aspergillus, Pichia, Cladosporium, Thermoascus, Xeromyces, Thermomyces, Monascus, Byssochlamys, Rasamsonia*, and *Rhizo* fungal genera varied during the brewing process (Figure 2D). For HDQ and MDQ, the relative abundances of *Aspergillus* and *Thermoascus* were >20%, representing the dominant fungal genera. The relative abundance of *Xeromyces* in MDQ was also >20% but different from that in HDQ, which may be related to various factors, such as the brewing crafts.

For LP, *Aspergillus, Pichia*, and *Byssochlamys* had a high relative abundance (all >18%), representing the dominant fungal genera in stacking-fermentation grains. The relative abundances of *Aspergillus, Pichia*, and *Thermoascus* in the FG (CA, CM, and CB) were >3%, representing the dominant fungal genera in FG. In conclusion, *Aspergillus, Thermoascus*, and *Pichia* were the most important fungal genera in different samples during the brewing process, which is consistent with the results of other studies (Wang et al., 2021; Cheng et al., 2023c; Xu et al., 2023).

### 3.2 Heat map, correlation network, and LEfSe analysis of microbial composition at the genus level

As shown in Figure 2E, different samples from the brewing process had no common dominant bacterial genera, with at least two bacterial genera exhibiting high relative abundances in different samples, including *Lactobacillus, Bacillus, Weissella, Leuconostoc*, and *Mitochondria*. *Bacillus* had a relatively high abundance in HDQ-1, HDQ-2, and HDQ-3, at 20.17%, 43.68%, and 44.03%, respectively (Figure 2E). Similarly, *Bacillus* had a high relative abundance in FQ-1, FQ-2, and FQ-3, at 5.96%, 37.16%, and 36.18%, respectively. Notably, the relative abundances of the bacterial genera *Methanosarcina, Clostridium_sensu_stricto_12, Caproiciproducens*, and *Syntrophomonas* were all high in the CP (CP-1, CP-2, and CP-3). This was significantly different from the other samples, indicating that the bacterial community composition of CP is unique at the genus level and that the microbial composition of CP includes anaerobic bacteria (Cheng et al., 2023d, 2024).

As shown in Figure 2F, no common dominant fungal genera were present in the different samples; however, at least two fungal genera with high relative abundances were present in different samples, including *Paecilomyces, Alternaria, Penicillium, Xerochrysium, Rhizopus, Mucor, Millerozym, Saccharomycopsis*, and *Lichtheimi*. Additionally, 12 fungal genera, including *Aspergillus* and *Xeromyces*, were mainly concentrated in CA, CB, and CP. The relative abundances of *Rasamsonia, Pichia*, and *Aspergillus* in CM were higher than those in other samples. In conclusion, the fungal communities of CA and CB affected those of CM, with noticeable differences at the genus level.

**Figure 3:**
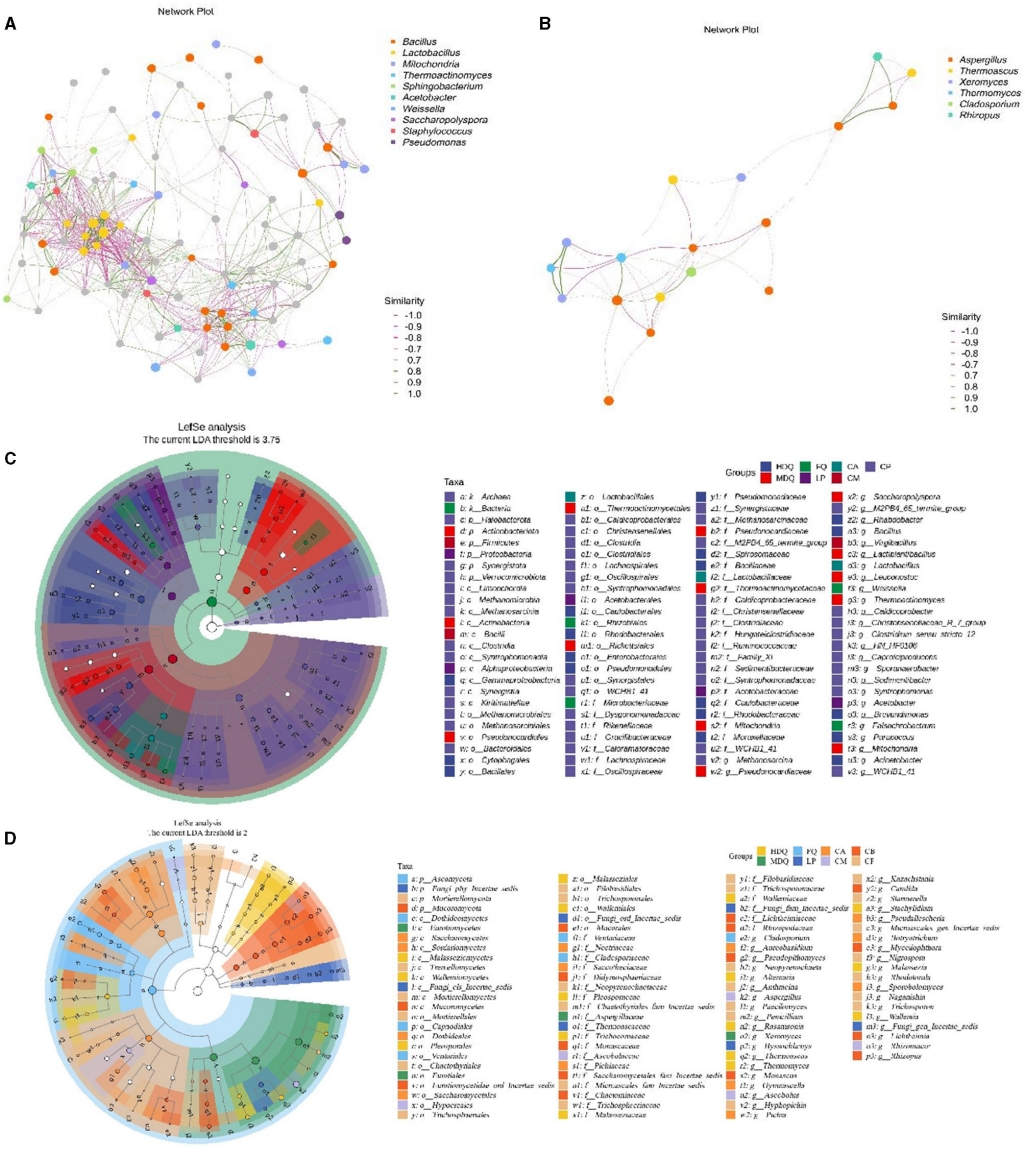
Correlation network and LEfSe analysis of the microbial community at the genus level between different samples during the brewing process. **(A)** hierarchical cluster of bacteria; **(B)** hierarchical cluster of fungi; **(C)** correlation network of bacteria; **(D)** correlation network of fungi; **(E)** LEfSe analysis of bacteria; **(F)** LEfSe analysis of fungi.

As shown in Figure 3A, *Bacillus, Lactobacillus, Weissella*, and *Staphylococcus* exhibited a strong positive correlation with other bacterial genera, representing the core genera during the brewing process. Moreover, a significant negative correlation was noted between *Bacillus* and *Lactobacillus* as well as between *Staphylococcus* and *Pseudomonas*. Additionally, *Aspergillus* and *Rhizopus* were positively correlated with other fungal genera (Figure 3B), representing the core fungal genera, which was consistent with the results shown in Figure 2B. Meanwhile, *Cladosporium, Thermoascus*, and *Aspergillus* were negatively correlated with each other.

As shown in Figures 3C, D, 325 bacterial genera were upregulated in CP, including *Clostridiales* and *Lactobacillales*; 33 fungal genera were upregulated in CP, including *Aspergillaceae, Mucoraceae*, and *Candida*. For LP, five bacterial genera were upregulated, with *Microlunatus, Haloplasma*, and *Ureibacillus* showing differences between groups. In addition, nine fungal genera were upregulated in LP, with several fungal genera, including *Scutellinia, Monocillium*, and *Thermobacillus*, exhibiting differences between groups.

In the samples from different stages of the brewing process, significant differences were observed in the Firmicutes and Bacillus phyla, the Ascomycota and Basidiomycota phyla, as well as the *Aspergillaceae* genus. Our findings were consistent with those of the previous studies, which have reported significant differences in abundances of the Firmicutes phylum and *Bacillus* genus of bacteria as well as the Ascomycota phylum and Aspergillaceae family of fungi between the FG and PM samples (Cheng et al., 2023a, 2024).

In conclusion, the bacterial genera *Bacillus, Lactobacillus, Weissella*, and *Staphylococcus*, as well as the fungal genera *Aspergillus* and *Rhizopus*, were the core microbial genera during the CFB brewing process, which is also consistent with the findings of previous studies (Cheng et al., 2023a, 2024).

### 3.3 Coordinate and traceability analyses of the microbial community in different samples during the CFB brewing process

The microorganisms in FG during Baijiu brewing mainly originated from the JQ, LP, and the brewing environment. However, the traceability analysis of microorganisms in FG lacks quantitative and systematic evaluation (Zou et al., 2018; Cheng et al., 2022). As a wide range of environmental microorganisms are involved in food fermentation, their exact contributions to the process remain unknown (Zou et al., 2018; Cheng et al., 2022; Tu et al., 2022). Therefore, tracing the microorganisms involved in Baijiu brewing is of great significance for controlling and improving its quality. Based on the CFB brewing process, we characterized the microbial communities of HDQ, MDQ, FQ, LP, FG (CA, CM, and CB), and CP. Further, the origin of microbial communities in the FG was revealed via FEAST traceability and correlation analysis.

#### 3.3.1 Coordinate and traceability analysis of bacterial communities in FG at the genus level

**Figure 4:**
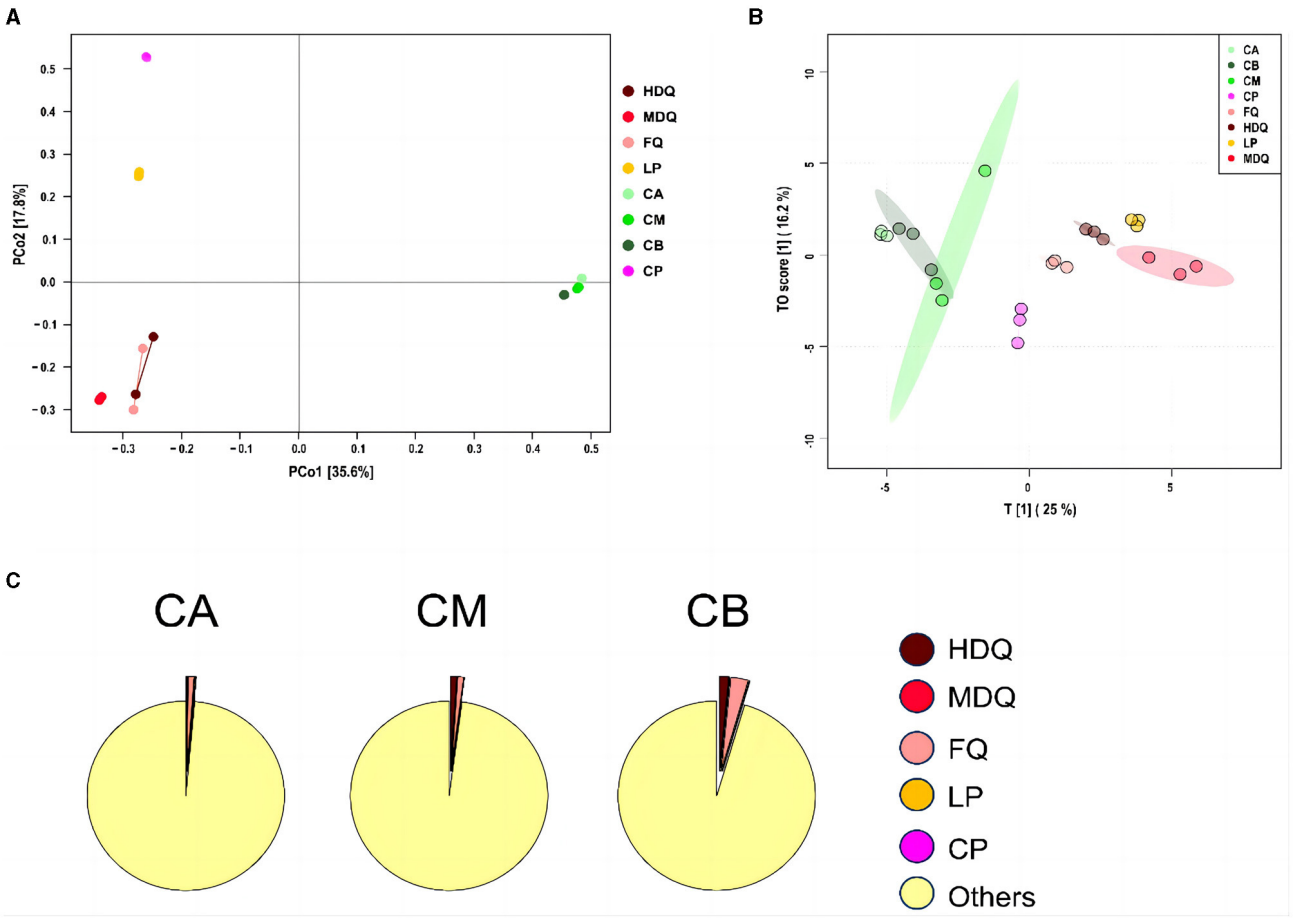
Coordinate and traceability analyses of the bacterial communities between samples during the brewing process. **(A)** PCoA; **(B)** OPLS-DA; **(C)** traceability analysis.

As shown in Figure 4A, the scores of principal components of the bacterial communities in each sample group were distributed in different regions, with the scores of principal components 1 and 2 being 35.6% and 17.8%, respectively. Among them, the HDQ and MDQ scores were close to each other in terms of the scores of principal components 1 and 2. In addition, CA, CM, and CB were close to each other, indicating considerable overlap among the bacterial communities at different levels of FG. The distances from LP and CP to the other samples were considerable, without any overlap area, indicating distinct bacterial flora of LP and CP.

As shown in Figure 4B, FQ, MDQ, LP, and CP were concentrated in similar areas, and their concentrations were high, indicating little interaction between the bacterial flora of these four sample groups. The area of CM was large, and it overlapped with those of CA and CB, indicating that the bacterial flora at different levels of FG (CA, CB, and CM) interacted with each other.

As shown in Figure 4C, most of the bacterial communities in the FG at different levels (CA, CM, and CB) originated from other sources (>95%). The bacterial communities in CA were 0.98% from FQ, 0.19% from LP, and 0.14% from HDQ. In addition, MDQ and CP contributed minimally to the bacterial origin of the CA (0.048% and 0.06%, respectively). Therefore, FQ and LP contributed significantly to the bacterial origin of CA. For CM, 1.05% of the bacterial community originated from HDQ, 0.96% from FQ, 0.04% from LP, 0.009% from MDQ, and 0.007% from CP, indicating that HDQ and FQ contributed more to the bacterial sources of CM, whereas LP, MDQ, and CP contributed less to the bacterial sources of CB.

For CB, 2.84% of the bacterial community originated from FQ, 1.54% from HDQ, 0.08% from LP, 0.05% from MDQ, and 0.02% from CP, indicating that FQ and HDQ contributed the most as bacterial sources for CB, whereas CP contributed less. In conclusion, bacterial sources with high contents of FQ and HDQ may have great potential in the fermentation of different levels of FG (Cheng et al., 2022). Daqu is the main source of strict and facultative aerobes in prokaryotic communities in FG (Wang et al., 2017).

Notably, environmental microorganisms on the surface of tools are the main contributors of bacterial and fungal communities in FG during heap fermentation and the beginning of pit fermentation (Lu et al., 2022). The environment plays an central role in tracing the origin of brewing microbial flora, and ecological monitoring of the brewing environment is of major relevance to Baijiu fermentation. The environmental microbial flora was closely related to the succession of microorganisms in the Baijiu fermentation ecosystem and FG, which are similar to those reported in the literature (Wang et al., 2017; Li et al., 2022).

#### 3.3.2 Coordinate and traceability analysis of the fungal community in FG at the genus level

**Figure 5:**
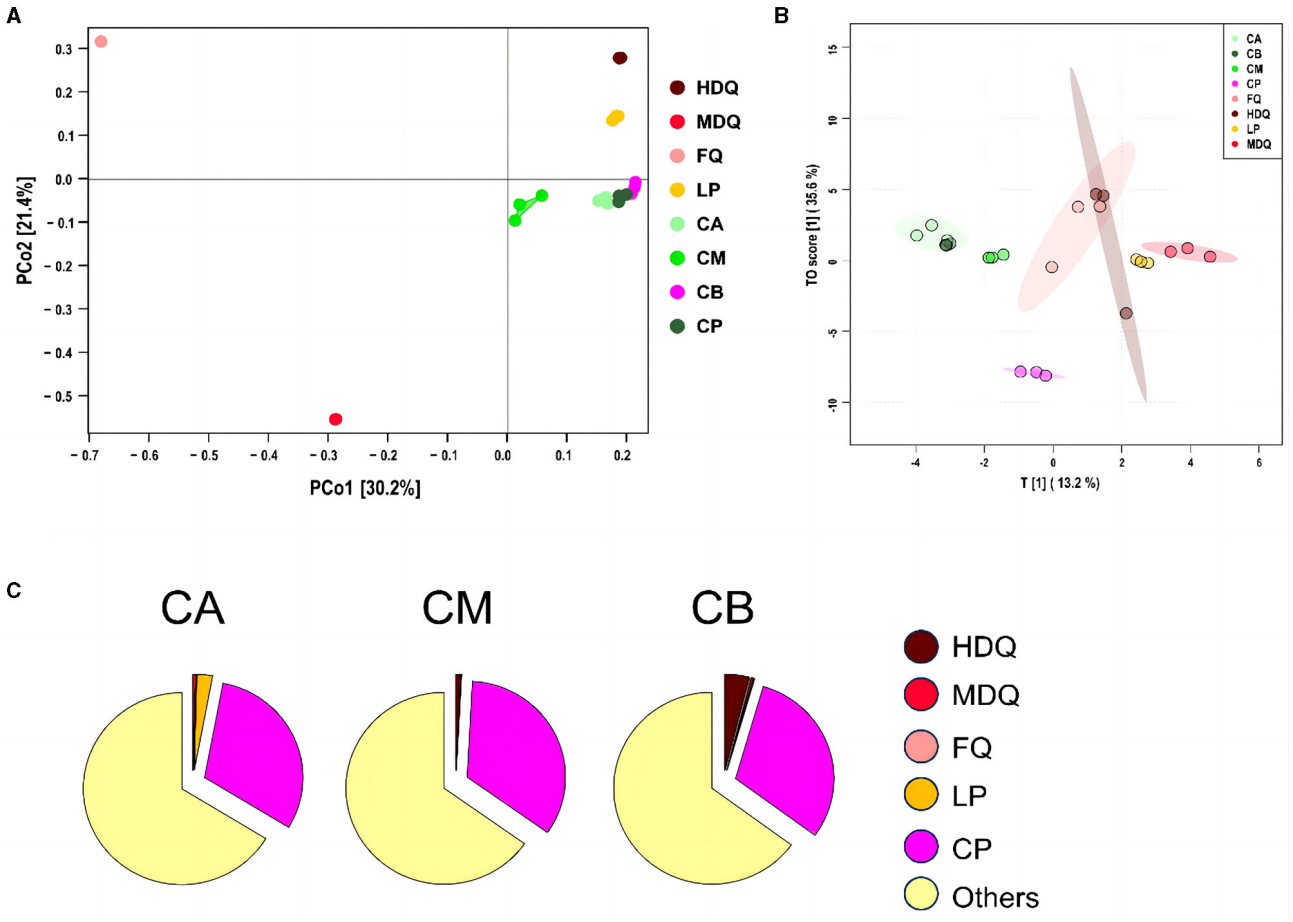
Coordinate and traceability analyses of the fungal communities between different samples during the brewing process. **(A)** PCoA; **(B)** OPLS-DA; **(C)** traceability analysis.

As shown in Figure 5A, the scores for principal components of the fungal communities in each sample group were scattered across different regions, with the scores of principal components 1 and 2 being 30.2% and 21.4%, respectively. The distances between and within the same sample groups of FG (CA, CM, and CB) and CP at different levels were relatively small, indicating similar and stable composition of fungal genera in these four different sample groups. In addition, the fungal diversity of different sample groups was relatively dispersed, indicating differences in fungal diversity among the sample groups.

As shown in Figure 5B, significant differences in the fungal genus composition were noted among different sample groups. CA, CB, and CM were concentrated in similar regions, and their concentrations were high, indicating the presence of mutual influence between the fungal flora at different levels of FG. Moreover, the areas of LP and FQ were large and overlapped, indicating an interaction between their fungal flora.

As shown in Figure 5C, most of the fungal communities in FG at different levels (CA, CM, and CB) were derived from other aspects (>64%), and the sources of fungal communities in other aspects were significantly lower than those of the bacterial communities (>95%). Meanwhile, 0.04% of the fungal communities in CA were derived from MDQ, 2.57% from LP, and 30.69% from CP. In addition, HDQ and FQ contributed little to the fungal origins of CA. These results indicate that LP and CP significantly contribute to the fungal origin of CA, whereas CA does not contact the CP at the bottom of the pool, which may be mainly influenced by the mud covering the cellar (Cheng et al., 2022, 2023a). For CM, 0.82% of the bacterial community originated from HDQ, 0.01% from LP, 0.02% from MDQ, and 33.93% from CP, indicating that CP contributed more to the fungal sources of CM, whereas FQ contributed less.

For CB, 4.00% of the fungal community originated from HDQ, 0.35% from MDQ, 0.18% from LP, and 30.52% from CP, indicating that HDQ and CP contributed relatively more to FQ. In conclusion, fungal sources with high CP content showed great potential for FG fermentation. Wang et al. (2017) reported that aerobic and anaerobic bacteria during the fermentation process mainly originated from Daqu, whereas anaerobic bacteria mainly originated from pit-mud. Therefore, environmental microorganisms, such as those in Daqu and CP, may contribute more to fermentation, which is consistent with our findings.

Our results revealed that environmental microorganisms were major contributors to the microbial traceability in FG of CFB, along with considerable differences in the microbial traceability between grains at different levels of fermentation (Wang et al., 2017). Therefore, the sampling of environmental microorganisms and the traceability analysis of microorganisms in FG require further study, such as through increased sampling of environmental microorganisms at key brewing process stages as well as at workshop sites, including raw materials, tools, ground, workshop air, and brewing water.

### 3.4 Analysis of TTMP content and traceability during the CFB brewing process

A standard curve for TTMP detection was constructed using an external standard method. The linearity was good over the mass concentration range of 2.0 to 50 μg/kg, with a linear regression equation of *y* = 1,057*x* and a correlation coefficient (R^2^) of 0.999 1 (Supplementary Figure S2), indicated that the standard curve exhibited good linearity and met the requirements for TTMP detection.

#### 3.4.1 Traceability of TTMP in JQ and LP

**Figure 6:**
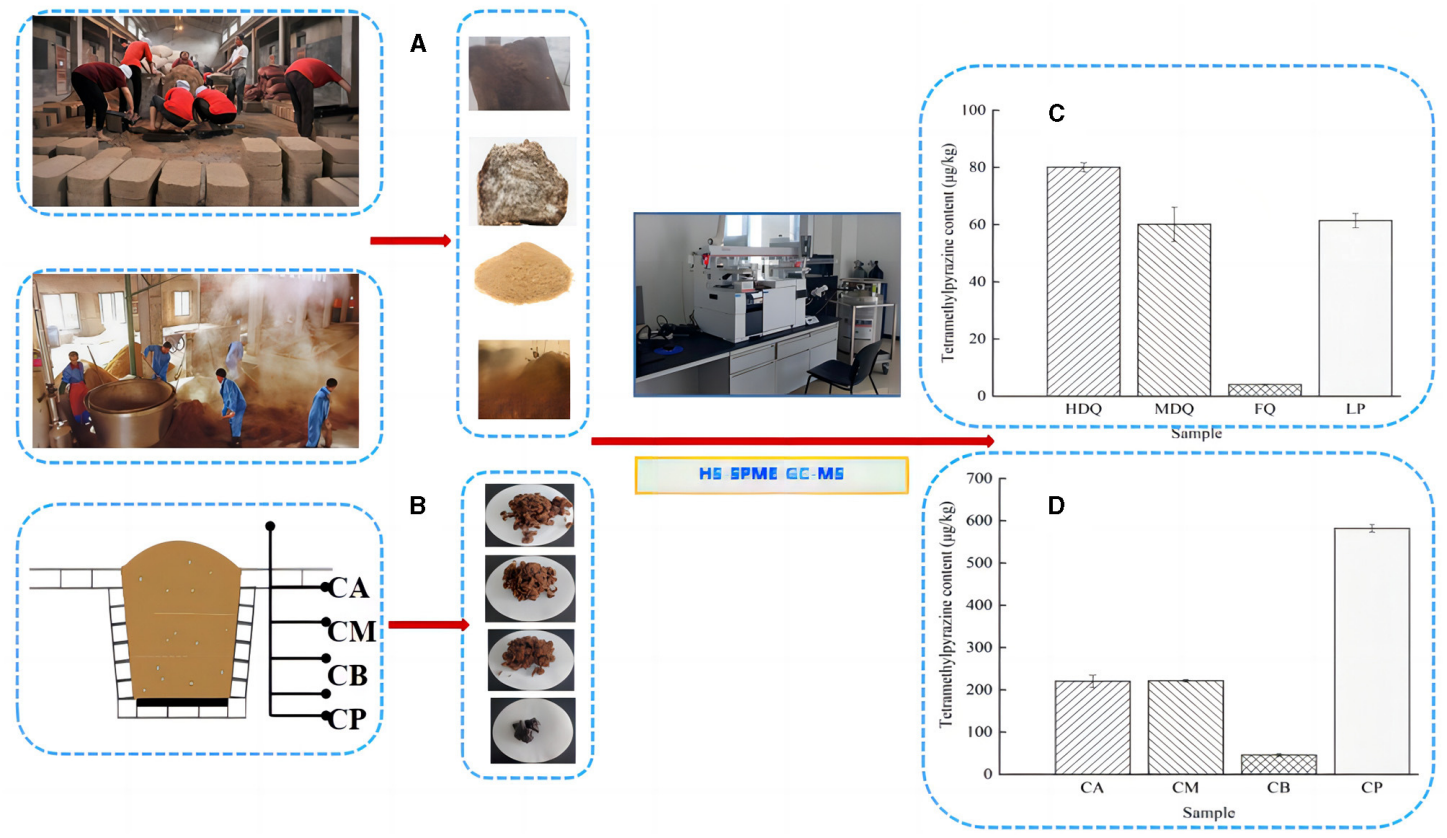
Sampling and comparison of TTMP content in different samples. **(A)** Sampling of JQ and LP; **(B)** sampling of FG and CP; **(C)** TTMP content of Jiuqu and LP; **(D)** TTMP content of FG and CP.

As shown in Figures 6A, C, Table 1, the TTMP content in HDQ (80.07 ± 1.51 μg/kg) was significantly higher than that in MDQ and FQ (60.10 ± 5.97 and 3.98 ± 0.16 μg/kg, respectively), indicating that HDQ was an important source of TTMP in LP. The Maillard reaction can accumulate pyrazine compounds for food production, which are beneficial for color and flavor (Jalbout and Shipar, 2007; Starowicz and Zieliński, 2019; Shi et al., 2022). The temperature of HDQ can reach 60–70°C, with the abundant amino and carbonyl compounds in the raw materials providing favorable conditions for the Maillard reaction (Cheng et al., 2022; Shi et al., 2022). Furthermore, non-enzymatic browning makes HDQ darker, giving it a more intense scorched and fried aroma (Starowicz and Zieliński, 2019; Cheng et al., 2022; Shi et al., 2022). Under suitable conditions, the production of Maillard reactants is positively correlated with the reaction temperature and duration (Starowicz and Zieliński, 2019; Shi et al., 2022), which may contribute to the higher TTMP content in HDQ than that in MDQ and FQ.

**Table 1:** The content of TTMP detection results in different samples (based on HS-SPME-GC-MS method).

**Samples**	**Moisture content (%)**	**Mean ±standard deviation**	**Samples**	**Moisture content (%)**	**Mean ±standard deviation**
HDQ	11.23	80.07 ± 1.51	CA	58.54	220.01 ± 14.69
MDQ	13.58	60.10 ± 5.97	CM	57.58	221.19 ± 2.97
FQ	6.18	3.98 ± 0.16	CB	63.24	45.32 ± 3.25
LP	45.59	61.41 ± 2.48	CP	53.79	581.91 ± 8.76

Isolating high-yield TTMP production strains and applying them in the brewing process to increase the TTMP content in Baijiu is a common technique (Jalbout and Shipar, 2007; Starowicz and Zieliński, 2019; Shi et al., 2022; Liu et al., 2023). Liu et al. (2023) isolated a strain of *Bacillus velezensis* with high production of TTMP from strong-flavor Baijiu, which could increase the content of TTMP in liquor by about 88%, and conjectured the mechanism of TTMP production in the strain by transcriptome sequencing analysis. As shown in Figure 6C, Table 1, the TTMP content in the FQ was 3.84 ± 0.01 μg/kg. FQ is typically produced by manually inoculating sterilized wheat bran with purebred microorganisms, such as bacteria, yeast, and *Aspergillus niger* (Cheng et al., 2022). The accumulation of TTMP in FQ mainly depended on bacteria, whereas the proportion of bacteria in FQ was relatively low (bacterial-FQ accounted for 10%, *Aspergillus niger*-FQ accounted for 70%, and yeast-FQ accounted for 20%), contributing to the low TTMP content in FQ.

The TTMP content of LP was 61.41 ± 2.48 μg/kg. The high-temperature stacking-fermentation of LP was beneficial to the Maillard reaction, which plays an important role in TTMP accumulation. Additionally, higher oxygen content in the LP contributes to the reproduction and metabolism of aerobic *Bacillus*, which in turn produces more TTMP. Notably, *Bacillus* predominates after the accumulation of grains during the CFB brewing process (Cheng et al., 2023c). *Bacillus* can produce multiple enzyme types, such as proteases, amylases, and cellulases, and the main metabolites of *Bacillus* include TTMP (Tu et al., 2022; Cheng et al., 2024).

#### 3.4.2 Traceability of TTMP in FG and CP

As shown in Figures 6B, D, Table 1, the TTMP contents in CA and CM were 220.01 ± 14.69 and 221.19 ± 2.97 μg/kg, respectively, significantly higher than that in CB (45.32 ±3.25 μg/kg). During the brewing process, in addition to the TTMP introduced by HDQ, MDQ, and FQ, TTMP accumulated during the stacking-fermentation of grains. TTMP also accumulated in the upper and middle grains during fermentation. The accumulated TTMP of FG at different levels varied, which may be due to the heat production of FG and the uneven heat transfer between the media, leading to temperature partitioning during brewing. Simultaneously, the environments of the upper, middle, and lower levels of the FG were different, which may have influenced the species enrichment and their metabolic activities, thereby influencing TTMP production and accumulation (Shi et al., 2022; Liu et al., 2023).

As an important part of the fermentation vessel, CP can provide flavor substances for Baijiu and plays an important role in the brewing of strong-flavor Baijiu and CFB (Cheng et al., 2022, 2024; Tu et al., 2022). As shown in Figure 6D and Table 1, the TTMP content in CP was 268.90 ± 4.05 μg/kg, and this high content may be attributed to the favorable anaerobic environment, as *Bacillus* and *Lactobacillus* are significantly correlated with the production of most volatile compounds in CFB (Hong J. et al., 2021; Cheng et al., 2023a, 2024). During fermentation, the temperature of CB was similar to that of CP, and the content of TTMP in CP was approximately 13 times that of CB (Figure 6D, Table 1). This may be attributed to the metabolism of abundant *Bacillus* species accumulated in CP over the years (Hu et al., 2015; Cheng et al., 2023a), producing a large amount of TTMP. However, parts of FG are distilled immediately after fermentation and leave the fermentation environment of the cellar (Cheng et al., 2022, 2023c; Xu et al., 2023).

In conclusion, HDQ was an important source of TTMP in LP. In addition to the TTMP derived from HDQ, MDQ, and FQ, TTMP accumulated during the stacking-fermentation of grains (LP) was also present in FG. Furthermore, TTMP in the top and middle levels of FG accumulated significantly during the fermentation process. CP also contributed to TTMP accumulation in the CB. Due to the insufficient understanding of the mechanism of Baijiu production, precise control of the fermentation progress, core brewing microorganisms, and characteristic flavor compounds could not be implemented (Zou et al., 2018; Wang, 2022; Xu et al., 2022; Pan et al., 2023). Valuable insights into the traceability of microbial communities and TTMP content during the brewing process presented in this study are beneficial for better understanding the mechanism and flavor profile of CFB. However, the influence of environmental microorganisms on microbial origins in FG was not addressed in our research, and further traceability analyses during CFB fermentation are warranted.

#### 3.4.3 Correlation analysis of microbial community and TTMP based on the brewing process of CFB

**Figure 7:**
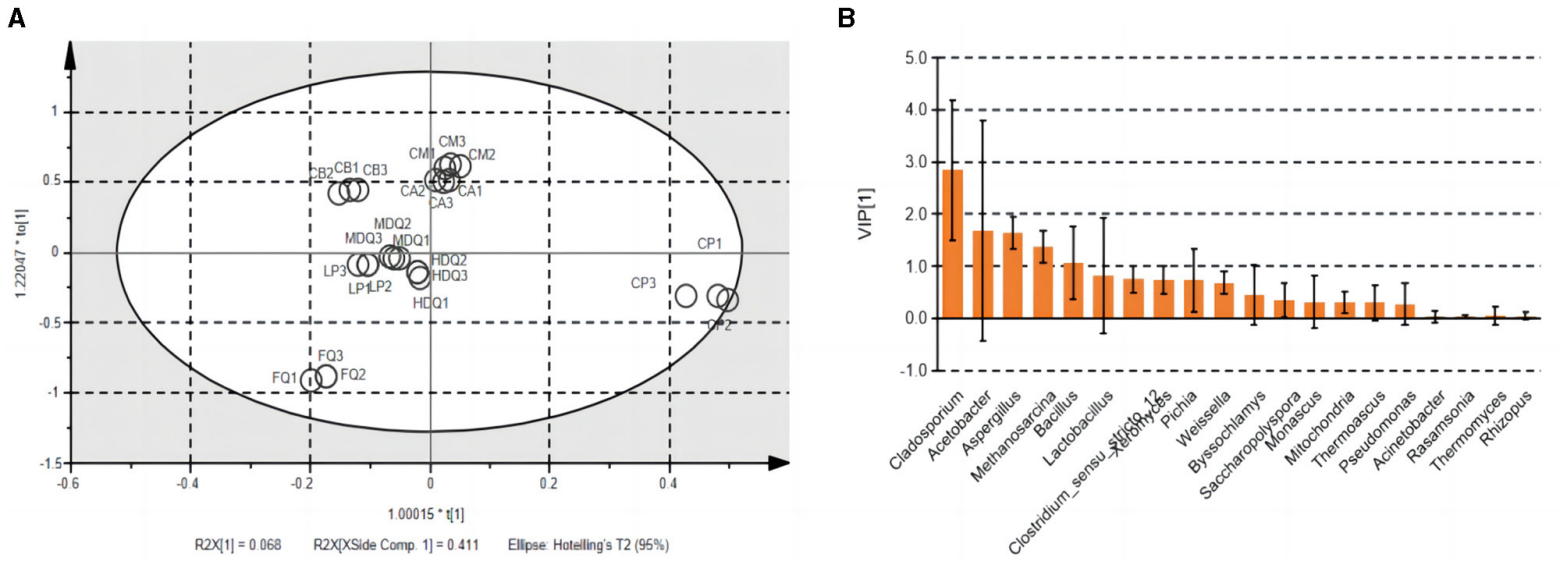
The scatter plot **(A)** and VIP plot **(B)** of microbial community correlated with TTMP based on OPLS in different samples during the brewing process.

Notably, TTMP was produced by microorganisms during the brewing process of CFB. Therefore, OPLS analysis was performed to calculate the VIP values for the microbial community vs. the TTMP to evaluate the core microorganisms producing TTMP (Figure 7) and the core volatile organic compounds (VOCs) conferred by microorganisms. The R^2^X, R^2^Y, and Q^2^ of the model were 0.969, 0.993, and 0.982, respectively, suggesting the model was well fitted for analysis and prediction. The scores of the scatter plot revealed that the samples were separated based on the microbial community and TTMP (Figure 7A). Further, the CP and FQ were both more diverse from the other samples, which may be due to the accumulation of *Bacillus* in the CP over the years, producing a large amount of TTMP, whereas the FG were distilled after the end of fermentation and thus left the fermentation environment of the pit (Cheng et al., 2022, 2023c).

The VIP plot summarizes the importance of variables to explain X and to correlate to Y, with a value of >1.0 indicating an important variable. The microorganisms with higher VIP values (>1) were *Cladosporium, Acetobacter, Aspergillus, Methanosarcina*, and *Bacillus* (Figure 7B). Dan et al. (2024) reported that the addition of *B. subtilis JP1* intensified microbial interactions in jiupei fermentation, consequently enhancing the production of volatile flavor compounds, such as heptanoic acid, butyl hexanoate and 3-methylthiopropanol in jiupei. In addition, a strain of *B. velezensis* with high TTMP production was screened and identified in strong-flavor Daqu, and the yield of TTMP was 29.83 μg mL^−1^, which increased the TTMP content in Baijiu by 88% (Liu et al., 2023). Herein, the enhanced application of the strain of *Bacillus* was beneficial to increase the TTMP content during the brewing process of Baijiu.

In CP used for three types of strong flavor Baijiu, Hu et al. (2015) identified a total of 28 closely related bacteria, including 15 *Clostridium* and 8 *Bacillus* species, and they found that Clostridium *kluyveri* N6 could produce caproic, butyric, and octanoic acids, which can react with ethanol to synthesize the corresponding ethyl esters. In 1962, Kosuge and Kamiya (1962) discovered that *Bacillus natto* can biosynthesise TTMP and isolated TTMP from Japanese natto. Notably, *Bacillus subtilis* is the main TTMP producing strain, along with *Corynebacterium glutamicum* and *Lactococcuslactis* subsp. *Lactis biovar. Diacetylactis FC1* also produces TTMP via fermentation (Zhu and Xu, 2010; Zhu et al., 2010; Shi et al., 2022).

## 4 Conclusion

This study describes the traceability of the origin of microbial communities and TTMP content distribution in the FG. The bacterial genera *Bacillus, Lactobacillus, Weissella*, and *Staphylococcus*, as well as the fungal genera *Aspergillus* and *Rhizopus*, were the core microbial genera during the brewing of CFB. However, the relative abundances of *Methanosarcina, Clostridium_sensu_stricto_12, Caproiciproducens*, and *Syntrophomonas* in CP were significantly higher than those in the other samples.

FEAST traceability analysis showed that most of the bacterial communities at different levels of FG originated from other sources (95%). Bacterial sources with high contents of FQ and HDQ showed great potential in the fermentation process at different levels of FG. Most of the fungal communities at different levels of FG originated from other sources (64%). Furthermore, fungal abundance during CP has great potential in the fermentation process at different levels of FG. Based on the above, this study highlights that the environmental microbiota is an important source of brewing microbiota, which is highly relevant to the microbial succession and metabolic profiles during the brewing process of CFB. HDQ was highlighted as an important source of TTMP, and the TTMP originating from the HDQ, MDQ, and FQ also accumulated during the stacking-fermentation of LP, whereas that from CA and CM accumulated during the brewing process. In addition, the VIP values of OPLS analysis conjectured that the *Cladosporium, Acetobacter, Aspergillus, Methanosarcina*, and *Bacillus* were the core microorganisms producing TTMP during the brewing process of CFB.

This study provides valuable insights into the traceability of the origin of microbial communities and TTMP content distribution in FG for refining brewing techniques and improving the flavor profile of CFB. For traceability of the origin of microbial communities in FG, future research should consider the important roles played by microorganisms within the brewing environment.

## Data availability statement

The datasets presented in this study can be found in online repositories. The names of the repository/repositories and accession number(s) can be found in the article/Supplementary material.

## Author contributions

WC: Writing – original draft, Writing – review & editing. WL: Conceptualization, Funding acquisition, Writing – review & editing. XC: Data curation, Writing – review & editing. XX: Data curation, Writing – original draft. HL: Data curation, Writing – original draft. HZ: Data curation, Writing – original draft. RL: Data curation, Writing – original draft. TP: Data curation, Software, Writing – original draft. NL: Data curation, Methodology, Software, Writing – original draft. HY: Data curation, Writing – original draft.
