# Supplementary figures and images for: Source and succession of microbial communities and tetramethylpyrazine during the brewing process of compound-flavor Baijiu
Source: Front Microbiol. 2024 Aug 6;15:1450997. doi: 10.3389/fmicb.2024.1450997 (PMC11333356; doi:10.3389/fmicb.2024.1450997)

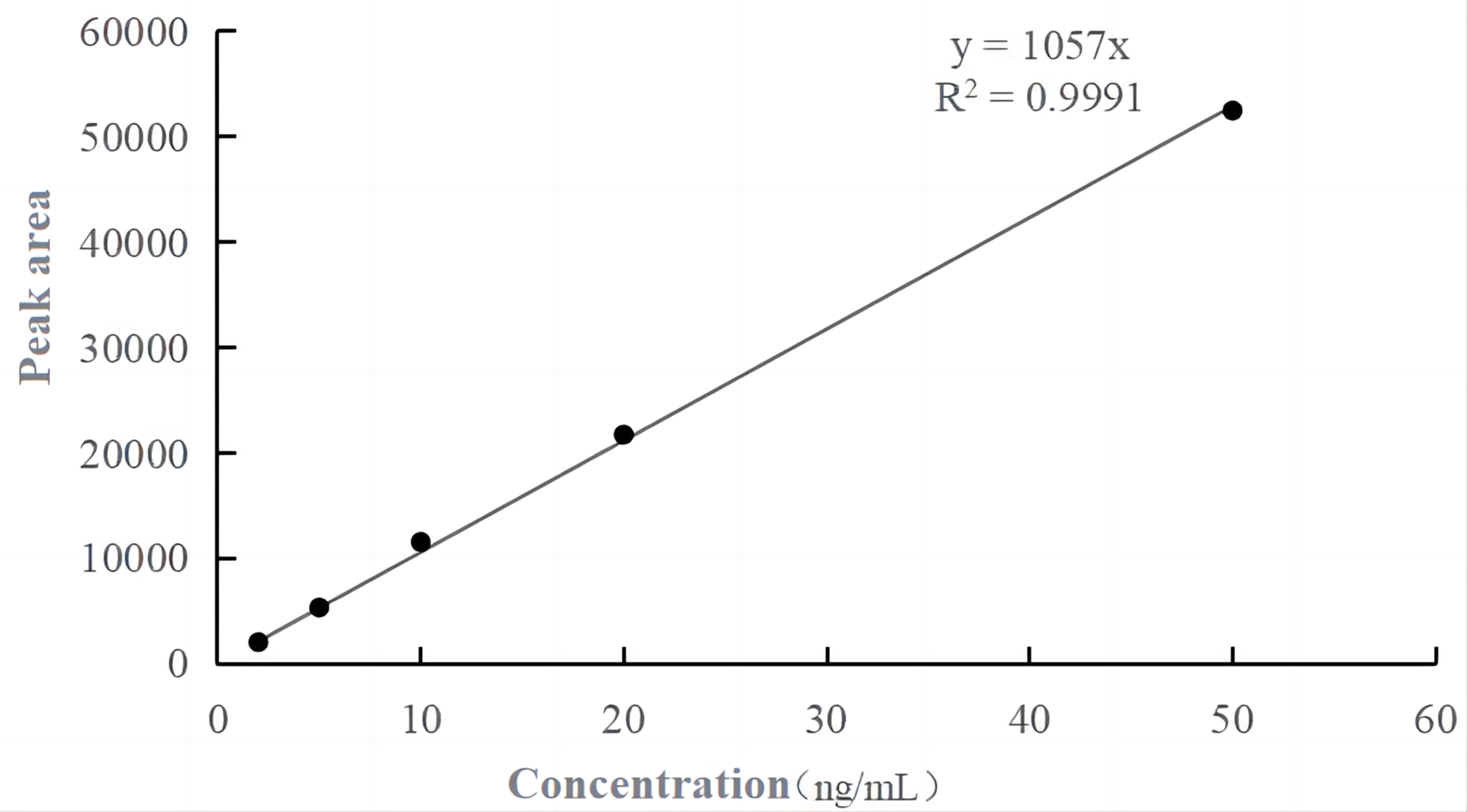

Supplement: Supplementary file 1 [file Image_1.JPEG]
